# Painkiller intake and problematic health literacy in sport and music students - A cross-sectional study

**DOI:** 10.1038/s41598-024-63127-3

**Published:** 2024-05-31

**Authors:** Katharina Pöppel, Gunter Kreutz, Maren Müller, Dirk Büsch

**Affiliations:** 1 School IV - School of Humanities and Social Sciences, Carl von Ossietzky Universität Oldenburg, Institute of Sport Science, Ammerländer Heerstraße 114–118, 26129 Oldenburg, Germany; 2 School III - School of Linguistics and Cultural Studies, Carl von Ossietzky Universität Oldenburg, Institute of Music, Ammerländer Heerstraße 114–118, 26129 Oldenburg, Germany

**Keywords:** Analgesics, University, Young adults, Online survey, Bayesian statistics, Health care, Risk factors

## Abstract

Previous works have suggested a high prevalence of painkiller intake (PI) among sport students but also improved health literacy (HL) for sports-active students than for sports-inactive students. Since health-related content also forms part of the sport science curriculum, the study focuses on these seemingly paradoxical results. Music students who are also physically active through their instrumental practice, who act in an area with increased PI and who have no health-related teaching content in their curriculum composed the comparison group. Therefore, this study investigated the prevalence of PI and HL in cohorts of sport (*n* = 222; 54.5% female) and music students (*n* = 89; 67.4% female) using a cross-sectional online survey in Lower Saxony, Germany. The hypothesis tests were validated by calculating frequentist and Bayesian statistics. The results show that 50.9% of sport and 28.1% of music students exhibit PI concerning their study programs, often for prophylactical purposes and in the presence of low HL levels. The weak negative correlation between PI and HL was not statistically confirmed and requires further research with improved test power. Regarding the possible health consequences of an inconsiderate PI, target group-specific prevention is indicated to increase general health awareness and HL.

## Introduction

Self-medication via painkillers is a widespread behavior internationally [e.g.,^1,2^] and is associated with significant health risks due to possible physical sequelae and side effects [e.g.,^3,4^]. A target group that is particularly noticeable in the context of painkiller intake (PI) is physically active people who rely on a functioning body as part of their career^5^. In addition to physically demanding professions, athletes and musicians are also included and build the underlying target group of this study. Pain and PI are prevalent not only in the upper-performance spectrum, like professional music (e.g., orchestra musicians) and competitive sports^6,7^. This issue also affects non-professionals, such as students in university education^8,9^. The data suggest that young people engage in widespread PI in areas that lead to later activities as multipliers of education, e.g., physical and health education teachers. This involves, for example, educating and supporting others in a health-competent lifestyle. Therefore, the present study pursues three objectives: (1) a survey of the current prevalence of PI among sport and music students, (2) a survey of the health-related capacity for action (health literacy, HL) among sport and music students, and (3) an examination of the association between PI and HL among sport and music students.

Notably, sport students in German-speaking regions report high levels of PI during their studies. Here, the relevance of practical exams is highlighted, and 56% reported PI in general^8^, which is comparable to the lifetime prevalence. In comparison, collegiate athletes in the United States reported a point prevalence between 20 and 28%^10^. Despite differences in the aforementioned prevalence period, PI seems common in this population. Additionally, PI appears to be more prevalent in sport students than in the general German population, with a four-week prevalence of 46%^11^, or in amateur sports, with a one-year prevalence of 51%^12^. Compared to elite sports, PI appears to be common or more common in sport students than in elite handball players (one-year prevalence: 56%;^13^) or elite soccer players (average one-year prevalence: 33%;^14^). Based on these data, sports students stand out due to a comparably more prevalent PI. Consequently, one should focus on the specifics of this target group and the causes of PI.

Compared with the music setting, few studies have examined PI by musicians. Taking a one-year prevalence into account, 49% of orchestral musicians report PI^6^. Compared with the prevalence in a sport setting mentioned before, musicians seem to take slightly less painkillers. However, the study situation suggests that performance situations, e.g., concerts, competitions, or practical sport exams during studies, favor PI [cf.^6,8,13,14^].

To better understand the PI-related situation, it is worth contrasting sports and music settings. Comparisons between athletes and musicians reveal similarities, such as long-term physical demands for both groups, and differences, such as shorter professional careers and better access to professional health care for athletes in a high-performance context^15,16^. Sports and music differ in their causes of pain. In sports, the natural causes of an enhanced need for painkillers are manifold and encompass events such as trauma, injury, or general training overload [e.g.,^8^]. In the musical setting, pain is generally caused by numerous constant repetitions or practice overload^6^. In this respect, there are fewer reasons, notably fewer acute reasons, for pain in the music setting.

The PI is not always linked to the sensation of pain. A more problematic type of PI is for prophylactic purposes, as dosages are not tailored to the specific pain sensation [e.g.,^4,7,17^]. In the case of pain-related or prophylactic PI, there is inconsistent control over the dosage of PI [e.g.,^12^]. Increased PI, whether in dosage or duration, represents a potential health risk [e.g.,^18^]. Additionally, incorrect or unreflective PI can cause long-term health effects, such as functional and structural damage to the musculoskeletal system or acute and long-term organ damage, particularly affecting the stomach, kidneys, liver, and heart^19^. In summary, PI is also closely linked to health.

Promoting health is considered relevant in both sport and music education contexts. While addressing health-related educational content seems to be a new idea in higher education music institutions^20^, sport studies are characterized by the consideration of health-related content in the curriculum [e.g.,^21,22^]. Thus, sport students should gain a theoretical and practical health-related capacity for action during their studies. The construct of health literacy (HL) can be associated with this idea. According to Sørensen et al. [^23^, p. 3], HL “is linked to literacy and entails people’s knowledge, motivation and competences to access, understand, appraise, and apply health information in order to make judgments and take decisions in everyday life concerning healthcare, disease prevention, and health promotion to maintain or improve quality of life during the life course.” Research suggests that sport activity is beneficial for young people’s overall HL. Thus, young people who are active in sports have better HL than inactive young people^24^. Additionally, study results suggest that HL promotion has a beneficial effect on the PI of college athletes^10^. In this respect, sport students should have an advantageous basis, and PI can be used as a concrete example of applying HL and reflective decision-making.

There is evidence to suggest that sports students exhibit increased PI, although their curricula cover health-related learning content. This seems paradoxical, as one would assume a negative relationship between PI and HL. Furthermore, it can be assumed that music students tend to take painkillers in the context of their studies, while their curricula forgo health-related content. According to the study objectives and the reported study results, it was hypothesized that: (H1) sport students take more painkillers than music students; and that (H2) sport students show better HL than music students. Since the connection between PI and HL cannot be clearly deduced, it is hypothesized that (H3) there is a relation between the two variables.

## Materials and methods

### Study design

The study was designed as a cross-sectional online survey. The cross-sectional design offers the advantage of providing a current and economic overview of the relevance of PI and the connection between PI and HL in the defined target group. The data sampling procedure was restricted to all universities in Lower Saxony, Germany, offering sport and/or music studies. The survey period started in November 2022 and ended in February 2023.

### Population

A total of 413 individuals began the online questionnaire, which included an informed consent statement. The final sample comprised 311 students (*n* = 222 sport students, *n* = 89 music students; 58.2% females), as only those participants who had completed at least 50% of the entire questionnaire and could be identified as sport or music students were included in the evaluation (see Table 1). The mean age of the participants was 23.61 years (*SD* = 3.59, range: 18–42 years), whereas sport and music students showed a comparable age structure, *t*(309) = 0.30, *p* = 0.76, *d* = 0.04, 1-β = 0.85.

**Table 1:** General and pain-related characteristics of the sample.

		Sport students (*n* = 222)	Music students (*n* = 89)
Gender	Male	45.0% (*n* = 100)	31.5% (*n* = 28)
	Female	54.5% (*n* = 121)	67.4% (*n* = 60)
	Non binary	0.5% (*n* = 1)	1.1% (*n* = 1)
Age (years)	*M* (*SD*)	23.65 (3.57)	23.52 (3.64)
	Range	18–42	19–39
Number of semesters	*M* (*SD*)	6.03 (3.57)	6.90 (4.22)
	Range	1–20	1–21
Number of practical classes in the last year	*M* (*SD*)	2.55 (1.99)	4.67 (3.06)
	Range	0–9	0–12
Indication of pain in the last seven days		59.5% (*n* = 132)	59.6% (*n* = 53)
Pain regions (regardless of pain intensity)	Head	19.4% (*n* = 43)	46.1% (*n* = 41)
	Neck or shoulders	38.3% (*n* = 85)	41.6% (*n* = 37)
	Arms or hands	23.0% (*n* = 51)	25.8% (*n* = 23)
	Trunk	19.9% (*n* = 42)	28.1% (*n* = 25)
	Leg or feet	41.0% (*n* = 91)	19.1% (*n* = 17)
	Other body regions	15.8% (*n* = 35)	19.1% (*n* = 17)
Number of painful body areas	*M* (*SD*)	1.56 (1.54)	1.80 (1.81)
Prophylactic PI	Before exams	55.0% (*n* = 44)	15.8% (*n* = 3)
	Before teaching	47.5% (*n* = 38)	36.8% (*n* = 7)

### Materials

The online questionnaire was administered using the online survey tool LimeSurvey^25^, which ensured complete anonymity for the participants. The survey included the following categories: (1) pain, including an indication of pain regions and the intensity of pain within the last seven days (4-point Likert scale; 0 = no pain, 3 = severe pain; adapted from the Regional Pain Scale^26^); (2) PI, including study-related intake in general and study-related intake within the last year (dichotomous items whether intake was conducted or not), preferred agents (multiple choices of the most common active ingredients), sources of information about painkillers (multiple choices of information sources), and prophylactic use (dichotomous response, whether prophylactic use was conducted before exams or teaching or not) [cf.^8,13,17^]; (3) HL (measured by the German short form of the European Health Literacy Survey Questionnaire HLS-EU-Q16 and evaluated by a sum score of 0–8 = inadequate, 9–12 = problematic and 13–16 = adequate HL^27,28^); and (4) study-specific data (e.g., number of practical classes) and demographic characteristics. This study was approved by the institutional ethics committee (Carl von Ossietzky Universität Oldenburg, Drs.EK/2023/013), in accordance with the Declaration of Helsinki. Informed consent was obtained from all participants at the beginning of the survey.

### Statistical analyses

The statistical analyses were performed using SPSS version 29.0^29^ and JASP version 0.18.1^30^. Due to the existing data structure, nonparametric tests were used to check for potential differences in PI (chi-square test supplemented by odds ratio [OR] as an effect size [H1]) and HL (Mann–Whitney test supplemented by *r* as an effect size [H2]) between sport and music students. Additionally, a point-biserial correlation was used to analyze the relationship between PI and HL (H3). Since the study aims to evaluate the PI in sport and music studies, testing the first hypothesis is considered multiple testing based on five individual tests [cf.^31^]. For this reason, the significance level was adjusted using Bonferroni correction to avoid false positive statistical test results, leading to a level of *p* = 0.01. Following the recommendations of Büsch and Loffing^32^, the alternative hypothesis was verified in relation to the null hypothesis and how many times more likely the alternative hypothesis was to be true than the null hypothesis by calculating the Bayes factor. Using additional Bayesian statistics, the significance of the results can be increased compared to traditional statistical methods. Additionally, post-hoc power calculations were performed using G*Power version 3.1.9.7. software^33^.

## Results

### Painkiller intake among students

Assuming a more prevalent PI among sport students [H1], we calculated the frequency of PI in the two student groups concerning intake in their studies in general (study-related prevalence) and within the past twelve months (one-year prevalence; see Table 2 for detailed information on the statistical tests). The PI prevalence results showed that, independent of the substance, sport students took significantly more painkillers during their studies (50.9%, *n* = 113) than did music students (28.1%, *n* = 25, *p* < 0.001, OR = 2.65). A comparable result was shown for the one-year prevalence: sport students (36.0%, *n* = 80) indicated a greater PI within the last year than did music students (21.3%, *n* = 19, *p* = 0.01, OR = 2.07). This means that, sport students have a more than two times greater risk of PI in the context of their studies than music students. The Bayesian statistics provided substantial (BF_10_ = 3.60 for the one-year prevalence) to decisive evidence (BF_10_ = 140.54 for the study-related prevalence) to support the validity of the alternative hypothesis H1 in both cases [cf.^34^].

**Table 2:** Differences in PI between sport and music students.

Substance	Test	Odds Ratio (OR) 90% CI	Power (1-β)	Bayes statistic (BF_10_)
Overall PI (study-related prevalence)	*χ*^*2*^(1, *N* = 311) = 13.39, *p* < .001	2.65 [1.70, 4.15]	.66	140.54
Previous academic year PI (one-year prevalence)	*χ*^*2*^(1, *N* = 311) = 6.32, *p* = .01	2.07 [1.28, 3.37]	.46	3.60
Ibuprofen	*χ*^*2*^(1, *N* = 311) = 8.46, *p* = .004	2.42 [1.46, 4.03]	.55	11.28
Paracetamol	*χ*^*2*^(1, *N* = 311) = 3.28, *p* = .07	2.43 [1.06, 5.54]	.48	0.49
Diclofenac	*χ*^*2*^(1, *N* = 311) = 2.26, *p* = .13	2.53 [0.88, 7.21]	.53	0.23

To conduct a differentiated hypothesis test for H1, a comparison of specific active agents that focused on the one-year prevalence and on the three most frequently named painkillers was performed: ibuprofen (29.9%, *n* = 93), paracetamol (10.6%, *n* = 33, and diclofenac (6.8%, *n* = 21). Moreover, sport students (34.7%, *n* = 77) tended to take significantly more ibuprofen than did music students (18.0%, *n* = 16, *p* = 0.004, OR = 2.42, BF_10_ = 11.28). On a descriptive level, sport students also took more paracetamol (12.6%, *n* = 28) than did music students (5.6%, *n* = 5). However, this difference was not confirmed statistically (*p* = 0.07, BF_10_ = 0.49), mainly focusing on the OR and the lower confidence bound, which indicates possible equality between the two groups [cf.^35^]. The same phenomenon was shown for diclofenac, indicating solely on a descriptive level that sport students (8.1%, *n* = 18) take more diclofenac than music students (3.3%, *n* = 3, *p* = 0.13, BF_10_ = 0.23). Therefore, in terms of active ingredients, strong evidence for a more prevalent PI in sport students, confirmed by the Bayes factor^34^, was found only for ibuprofen.

### Health literacy of students

A sobering picture emerges for the HL of the sport and music students. Here, 75.2% (*n* = 161) of the sport students and 79.3% (*n* = 69) of the music students indicated inadequate HL (see Table 3). A comparison of the two student groups (H2) revealed that sport students (median, short *Mdn* = 10.00, mean absolute deviation, short *MAD* = 2.00) did not exhibit greater HL than did music students (*Mdn* = 10.00, *MAD* = 2.00; *U* = 8857.50, *p* = 0.25, 1-β = 0.49, BF_10_ = 0.23). Furthermore, the Bayes factor confirmed the equality of sport and music students’ HL, providing strong evidence to support the null hypothesis [cf.^34^].

**Table 3:** Comparison of HL levels between sport and music students (based on Röthlin et al.^27^).

HL level	Sport students	Music students
inadequate (0–8)	28.0% (*n* = 60)	31.0% (*n* = 27)
problematic (9–12)	47.2% (*n* = 101)	48.3% (*n* = 42)
adequate (13–16)	24.8% (*n* = 53)	20.7% (*n* = 18)

### Combined consideration of painkiller intake and health literacy

Looking at the relationship between the last year’s PI and HL (H3), sport students showed a nonsignificant negative correlation between PI and HL: *r*_*pb*_ = − 0.11, 90% CI [− 0.02, > − 0.99], *p* = 0.06, 1-β = 0.52. A comparable picture emerged for music students: *r*_*pb*_ = 0.06, 90% CI [− 0.08, > 0.99], *p* = 0.28, 1-β = 0.49. Therefore, the relationship between PI and HL cannot be confirmed here.

### Further outcomes

After further calculations were conducted and the association between study progress and HL was taken into account, the sport students indicated a weak positive correlation between study progress and HL: *r*_*s*_ = 0.20 [95% CI: 0.08, > 0.99], *p* = 0.004, 1-β = 0.60. Interestingly, there was also a weak positive correlation between study progress and HL among music students: *r*_*s*_ = 0.27 [95% CI: 0.14, > 0.99], *p* = 0.005, 1-β = 0.49.

Additionally, outside of hypothesis testing, the information provided by the students showed that more than half of the students (59.5%, *n* = 132 of the sport students and 59.6%, *n* = 53 of the music students) suffered from acute pain during the period surveyed (see Table 1). Most of the sport students (50.5%, *n* = 112) and almost half of the music students (49.4%, *n* = 44) reported pain in at least two body regions. There were differences in frequency and intensity between the sport and music students regarding the identified pain regions. While the sport students suffered comparatively more often and more intensely from pain in their legs and feet (*W* = 5074.00, *p* < 0.001, *r* = 0.45, 90% CI [0.32, 0.57]), the music students reported comparatively more headaches (*W* = 1865.50,* p* < 0.001, *r* = 0.48, 90% CI [0.34, 0.58]). Focusing on PI outside of hypothesis testing, prophylactic PI before exams was reported more by sport students (55.0%, *n* = 44) than music students (15.8%, *n* = 3, *χ*^*2*^(1, *N* = 99) = 9.47, *p* = 0.002, OR = 6.54, 90% CI [2.17, 19.61]).

## Discussion

PI is a prevalent behavior for sport and music students, as more than half of sport students and more than a quarter of music students use painkillers during their studies. The PI prevalence of sports students is in line with the findings of the data collection from 2017 by Bumann et al.^8^ and supplements it with a more precise one-year prevalence. Although the one-year prevalence is lower, PI and pain can be considered as a frequent side effect of sports studies. The data clearly show that taking painkillers is common even in the absence of pain.

Information on prophylactic use aggravates the extent of PI, particularly among sport students. Compared to findings on athlete behavior before sports competitions^4^, sport students showed event-related (e.g., exams) overuse of painkillers prior to the onset of pain. It is particularly striking that 47.5% of sport students take painkillers before regular teaching and thus in comparably low-threshold settings. Interpreting this result, students might feel under pressure at an early stage that breaks could jeopardize their study progress. In this context, Bumann et al.^8^ show indicators for a cost–benefit analysis in the form of individual qualitative statements on dealing with pain. Whether this assumption can be applied to prophylactic PI needs to be tested in follow-up studies. Prophylactic PI can be considered particularly risky for health since there is no physical sensation to reference during intake. Thus, the signals that indicate limits of physical performance might be suppressed or easier to ignore [cf.^4^]. Therefore, these results can be considered indicators of inconsiderate painkiller use, particularly among sport students and a sufficient health-related capacity for action seems even more important.

A general survey of the HL led to a sobering result. Although one would attribute an advantage to sport students compared to music students regarding their HL based on the integration of health-related content in the sports curriculum [cf.^21,22^], both student groups indicate severe deficits. Since painkillers can lead to severe short-term and long-term physical damage if used without reflection^4,19^, unreflective use can be regarded as problematic and hazardous to health. Therefore, future studies should also include the individual problem awareness of PI. Without awareness of potential adverse health sequelae, this information can be used as a further indicator of insufficient HL.

There is weak evidence that HL increases throughout studies. Looking at the statistical data, we find a low positive correlation, and a large confidence interval accompanies the correlation coefficient. Therefore, these results must be interpreted carefully. Additionally, this effect does not apply specifically to sport students or their curriculum since a similar correlation can be found for music students. In this respect, studying sports specifically does not seem to promote HL. Interpreting this result, there could be an issue with the sender (lecturer) or the receiver (sport student). On the sender side, health-related content is potentially not conveyed sufficiently, and/or the recipient may not see personal relevance. Curricular analyses in the context of school teaching research indicate a difference between curricula and practical implementation in the sport teaching-learning context^36^. Transferring this idea to a university setting is worth considering the existing inclusion of health-related learning content in teaching, which also considers the student's perspective. Although the data indicate a comparable initial level of HL, sport and music students differ in the causes of their pain and their PI behavior. Thus, subject specificity should be considered in the design of teaching-learning processes to support students in a more reflective PI and their HL [cf.^10^]. An HL-based decision of whether to take painkillers could require a consciously guided reflection process. The data can be interpreted as students downplaying PI or overestimating their physical resistance. In addition, their already low HL may not have been activated in the decision-making process. HL can be viewed as the result of an educational process since it would have to increase, for example, with increasing knowledge of health-related content or the motivation to address health-related topics in a self-referential manner [cf.^23^]. In this context, it makes sense to relate findings on optimistic self-referential errors about the harmfulness of smoking in young people^37^ to sport and music students and painkillers.

A correlation between HL and PI cannot be shown statistically. Instead, a diffuse picture emerges, which must be discussed methodically. The results can be interpreted in two directions: (1) HL and PI are primarily independent, or (2) the operationalization of HL using a general HL questionnaire (HLS-EU-Q16^27^) was too unspecific and not adequate for the setting of sport or music studies and painkillers. Therefore, an area-specific operationalization of HL or related constructs should be discussed. It would be conceivable to have a painkiller-focused subscale that goes beyond the use of medication—as mentioned in the HLS-EU 16^27^—so that the overall construct is represented in its breadth and is supplemented by a painkiller-specific interpretation. Additionally, the dose and specific intake indication represent essential information needed to better understand the PI of sport and music students. In addition, a general query about the perceived relevance of health-related topics for young target groups appears to be appropriate.

From a research methodological point of view, the results must also be critically classified. Despite validating the results using the Bayes factor, the statistical analyses are characterized by low test power. The present sample can be considered too small for detecting effects, so replication with a larger sample based on an a priori calculation of the required sample size is necessary. Nevertheless, considering the descriptive data, it is evident that PI and HL among sport and music students are problematic and should be further investigated.

## Conclusion

Numerous students carelessly take painkillers and appear to be unaware of side effects and consequential physical damage. Additionally, we observed an openness to prophylactic PI, indicating an underestimation of the adverse effects of painkillers. Considering the poorly reflected PI, particularly among sport students, constructive and subject-specific painkiller prevention seems to be necessary. The goal of painkiller prevention would be a reduced and reflected use, as abstinence seems utopian. Prevention measures should take different starting points into account. On the one hand, this prevention approach would have to be suitable for people who need to be made aware of the consequences of unreflective and excessive PI. On the other hand, people who are aware of the health risks of excessive intake but who have no alternative course of action should also benefit. Increasing health-competent behavior could be seen as access. Despite the weak correlation between HL and PI, improving HL or related constructs could lead to a more reflective PI. To strengthen the compliance of young students, queries of needs and interests should be carried out in advance. The aim of all these efforts should be to enable students to study as painlessly and injury-free as possible and, in the event of pain, to conduct a health-friendly pain management.

## Data Availability

The datasets that are used in the current study are not publicly available. Access will be granted by the corresponding author upon request.

## References

[1] Atzendorf J (2019). The use of alcohol, tobacco, illegal drugs and medicines — An estimate of consumption and substance-related disorders in Germany. Dtsch. Arztebl. Int..

[2] Mehuys E (2019). Self-medication with over-the-counter analgesics: A survey of patient characteristics and concerns about pain medication. J. Pain.

[3] Sinatra R (2010). Causes and consequences of inadequate management of acute pain. Pain Med..

[4] Küster M (2013). Consumption of analgesics before a marathon and the incidence of cardiovascular, gastrointestinal and renal problems: A cohort study. BMJ Open.

[5] Choi B (2020). Opioid use disorder, job strain, and high physical job demands in US workers. Int. Arch. Occup. Environ. Health.

[6] Paarup HM (2011). Prevalence and consequences of musculoskeletal symptoms in symphony orchestra musicians vary by gender: A cross-sectional study. BMC Musculoskeletal Disord..

[7] Berrsche G, Schmitt H (2022). Pain prevalences and analgesic use in junior athletes — A recent narrative review. Ger. J. Sports Med..

[8] Bumann A, Banzer W, Fleckenstein J (2020). Prevalence of biopsychosocial factors of pain in 865 sports students of the DACH (Germany, Austria, Switzerland) region — A cross-sectional survey. J. Sports Sci. Med..

[9] Kok LM (2016). The occurrence of musculoskeletal complaints among professional musicians: A systematic review. Int. Arch. Occup. Environ. Health.

[10] Christopher S (2020). Epidemiological profile of pain and non-steroid anti-inflammatory drug use in collegiate athletes in the United States. BMC Musculoskeletal Disord..

[11] Lange, C., et al. *Anwendung rezeptfreier Analgetika in Deutschland. Eine Befragungsstudie zum Anwendungsverhalten, zur Kenntnis von Anwendungsinformationen und Bewertung eines möglichen Packungsaufdrucks in der erwachsenen Allgemeinbevölkerung [Use of over-the-counter analgesics in Germany. A survey study on application behavior, knowledge of application information and evaluation of possible packaging printing in the general adult population]*. 2014; Available from: https://www.bundesgesundheitsministerium.de/fileadmin/Dateien/5_Publikationen/Drogen_und_Sucht/Berichte/Abschlussbericht-RKI-II.pdf.

[12] Hager L (2021). Sex differences in the consumption of over-the-counter analgesics among amateur volleyball players. BMC Sports Sci. Med. Rehabilit..

[13] John JM (2023). Prevalence of sport-related analgesic use in German elite handball players. Ger. J. Sports Med..

[14] Trinks S (2021). Declaration of analgesics on doping control forms in german football leagues during five seasons. Ger. J. Sports Med..

[15] Nygaard Andersen L, Roessler KK, Eichberg H (2013). Pain among professional orchestral musicians: A case study in body culture and health psychology. Med. Probl. Perform. Artists.

[16] Stanhope J (2016). Physical performance and musculoskeletal disorders: Are musicians and sportspeople on a level playing field?. Perform. Enhanc. Health.

[17] Overbye M (2020). Walking the line? An investigation into elite athletes’ sport-related use of painkillers and their willingness to use analgesics to train or compete when injured. Int. Rev. Sociol. Sport.

[18] Matava MJ (2016). Ethical considerations for analgesic use in sports medicine. Clin. Sports Med..

[19] Leyk D (2023). Analgesic use in sports. Results of a systematic literature review. Deutsches Ärzteblatt.

[20] Matei R, Ginsborg J (2022). Health education for musicians in the UK: A qualitative evaluation. Health Promot. Int...

[21] Krüger M, Emrich E, Güllich A, Krüger M (2013). Die Wissenschaft vom Sport. Sport - Das Lehrbuch für das Sportstudium.

[22] Siedentop D, van der Mars H (2022). Introduction to Physical Education, Fitness, and Sport.

[23] Sørensen K (2012). Health literacy and public health: A systematic review and integration of definitions and models. BMC Public Health.

[24] Paakkari L (2017). Health literacy and participation in sports club activities among adolescents. Scand. J. Public Health.

[25] LimeSurvey GmbH. *LimeSurvey: An Open Source survey tool*. Available from: http://www.limesurvey.org.

[26] Häuser W (2010). Validierung der deutschen Version der regionalen Schmerzskala zur Diagnose des Fibromyalgiesyndroms [Validation of the German version of the regional pain scale for the diagnosis of fibromyalgia syndrome]. Der Schmerz.

[27] Röthlin, F., J. Pelikan, and K. Ganahl. *Die Gesundheitskompetenz der 15-jährigen Jugendlichen in Österreich*. Abschlussbericht der österreichischen Gesundheitskompetenz Jugendstudie im Auftrag des Hauptverbands der österreichischen Sozialversicherungsträger (HVSV) [The health literacy of 15-year-old adolescents in Austria. Final report of the Austrian Youth Health Literacy Study on behalf of the Main Association of Austrian Social Insurance Institutions] 2013 2021–05–03]; Available from: https://www.sozialversicherung.at/cdscontent/load?contentid=10008.715507&version=1395738807.

[28] Sørensen K (2013). Measuring health literacy in populations: Illuminating the design and development process of the European health literacy survey questionnaire (HLS-EU-Q). BMC Public Health.

[29] IBM Corp., *IBM SPSS Statistics for Windows, Version 29.0*. 2022, Armonk, NY: IBM Corp.

[30] JASP Team, *JASP (Version 0.17) [Computer software]*. 2023.

[31] Bender R, Lange S (2001). Adjusting for multiple testing — When and how?. J. Clin. Epidemiol..

[32] Büsch D, Loffing F (2023). Interpretation of empirical results in intervention studies: A commentary and kick-off for discussion. Ger. J. Exerc. Sport Res..

[33] Faul, F., et al. *G*Power. Statistical Power Analyses for Mac and Windows*. 2020 2021–12–27]; Available from: https://www.psychologie.hhu.de/arbeitsgruppen/allgemeine-psychologie-und-arbeitspsychologie/gpower.

[34] Wetzels R (2011). Statistical evidence in experimental psychology: An empirical comparison using 855 t tests. Perspect. Psychol. Sci..

[35] Cumming G (2013). The new statistics: Why and how. Psychol. Sci..

[36] Ruin S, Stibbe G (2021). Health-oriented ‘Bildung’ or an obligation to a healthy lifestyle? A critical analysis of current PE curricula in Germany. Curric. J..

[37] Arnett JJ (2000). Optimistic bias in adolescent and adult smokers and nonsmokers. Addict. Behav..

